# *Mycobacterium avium* pleuritis with multiple nodules in the pleura

**DOI:** 10.1002/rcr2.608

**Published:** 2020-06-28

**Authors:** Hiroaki Ogata, Eiji Harada, Tomoaki Takao, Kayo Ijichi, Naoki Hamada, Koichiro Matsumoto

**Affiliations:** ^1^ Research Institute for Diseases of the Chest, Graduate School of Medical Sciences Kyushu University Fukuoka Japan; ^2^ Department of Respiratory Medicine National Hospital Organization Fukuoka National Hospital Fukuoka Japan; ^3^ Pathophysiological and Experimental Pathology, Department of Pathology, Graduate School of Medical Sciences Kyushu University Fukuoka Japan

**Keywords:** Epithelioid cell granuloma, *Mycobacterium avium*, nontuberculous mycobacteria, pleuritis, thoracoscopy

## Abstract

As opposed to tuberculosis, pleurisy hardly develops in patients with nontuberculous mycobacteria (NTM) infection. In spite of increasing prevalence of NTM infection, little is known about thoracoscopic or pathological findings of the NTM‐infected pleura. We now report the first case of NTM pleuritis with multiple granulomatous nodules in the pleura. A 74‐year‐old woman was admitted to our hospital due to massive effusion of the left thoracic cavity. The analysis of pleural fluid showed lymphocytic exudative effusions with increased levels of adenosine deaminase, although culture of the pleural fluid was negative. The patient accordingly underwent thoracoscopy, which revealed multiple pleural nodules. Biopsy of the nodules demonstrated epithelioid cell granulomas without caseous necrosis. In addition, culture of the biopsy specimens confirmed infection by *Mycobacterium avium*. As culture of pleural fluid often fails to detect NTM pathogens, demonstration of pleural nodules during thoracoscopy can contribute to prompt diagnosis and treatment of NTM pleuritis.

## Graphical abstract

Pleurisy hardly develops in patients with nontuberculous mycobacteria (NTM) infection. We report the first case of NTM pleuritis with multiple granulomatous nodules in the pleura.

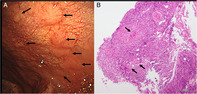

## Introduction

In contrast to tuberculosis, pleurisy hardly develops in patients with nontuberculous mycobacteria (NTM) infection [1]. Although the prevalence of NTM infection is remarkably increasing worldwide [2], little is known about thoracoscopic or pathological findings of the NTM‐infected pleura. Here, we describe a case of *Mycobacterium avium* (*M. avium*) infection with multiple nodules in the pleura at thoracoscopy.

## Case Report

**Figure 1:**
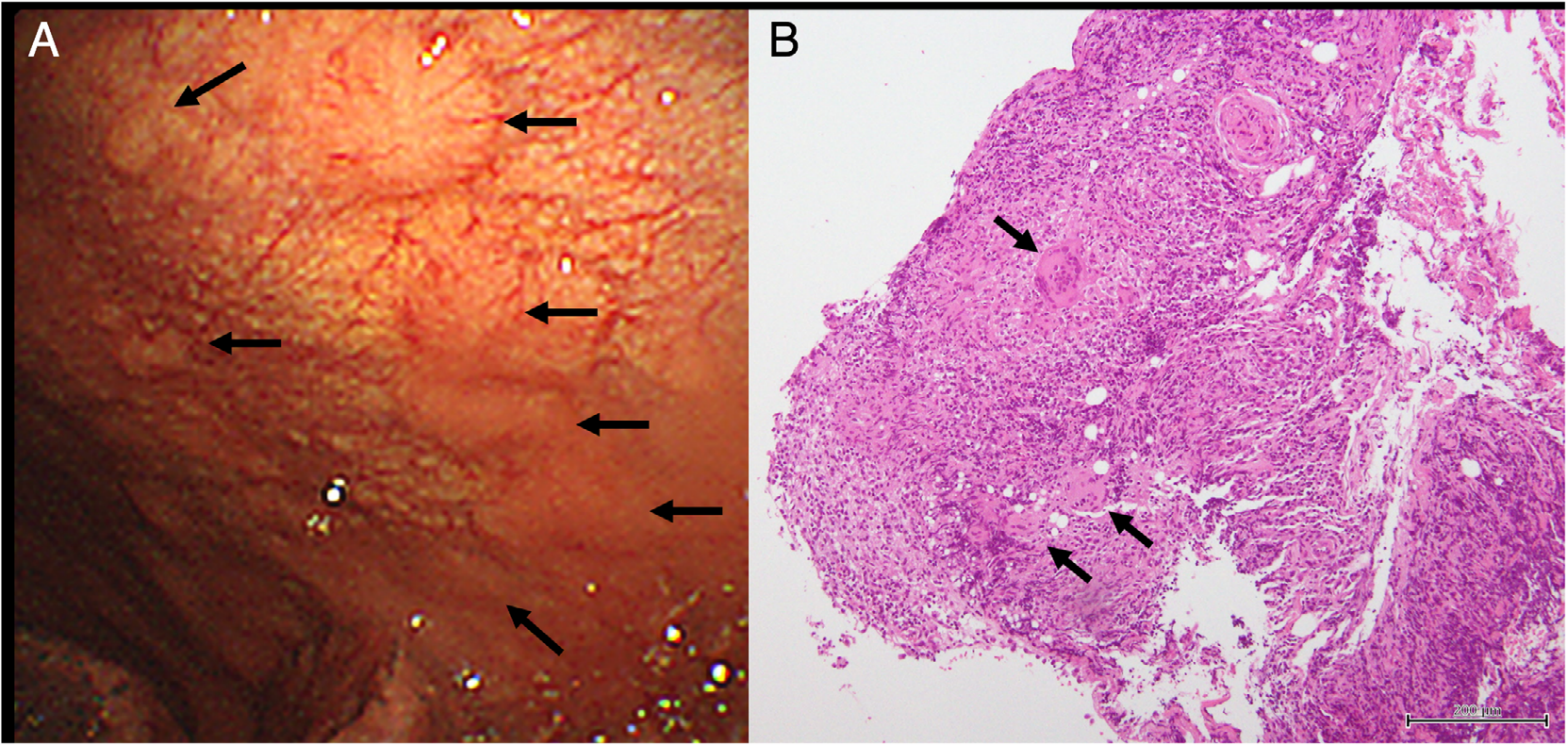
Thoracoscopic finding of the pleura. (A) Macroscopically, several nodules were observed (arrows). (B) Microscopically, epithelioid granulomas with multinucleated giant cells (arrows) were seen. There was no evidence of caseous necrosis (haematoxylin–eosin staining).

A 74‐year‐old Japanese woman presented to our hospital for progressive dyspnoea on exertion. Respiratory sounds were diminished on the left side, and the chest X‐ray revealed massive effusion of the left thoracic cavity. Blood examination detected anti‐*M. avium* complex antibodies (16.0 U/mL), and showed subtly elevated levels of C‐reactive protein (2.17 mg/dL) and glycosylated haemoglobin (6.7%). Both QuantiFERON TB Gold In‐Tube (QFT‐3G; Qiagen Inc., Germany) and serum interferon‐gamma neutralizing autoantibodies were negative. HIV antibodies were negative, whereas human T‐cell lymphotropic virus type I antibodies and circulating flower cells were positive, which led to the diagnosis of indolent adult T‐cell leukaemia‐lymphoma [3]. The analysis of aspirated pleural fluid revealed lymphocytic exudative effusions (95.0% of the total white blood cell count) with total protein levels of 5.3 g/dL, lactate dehydrogenase levels of 217 IU/L, and adenosine deaminase levels of 156.6 IU/L, although culture of the pleural fluid was negative. The patient underwent uniportal thoracoscopy through which multiple pleural nodules were observed (Fig. 1A). Pathologically, biopsy of the nodules demonstrated epithelioid cell granulomas without caseous necrosis (Fig. 1B). Furthermore, in spite of negative Ziehl–Neelsen smears, culture of the biopsy specimens confirmed infection by *M. avium*. After pleural fluid drainage, computed tomography of the chest revealed the nodular bronchiectatic form, and the culture of bronchial washing fluid was also positive for *M. avium*.

Considering the possibility of simultaneous infection of *Mycobacterium tuberculosis* and *M. avium*, the patient was initially treated as tuberculosis with isoniazid, rifampicin (RFP), pyrazinamide, and ethambutol (EB). Culturing all the above‐mentioned samples for six weeks, the patient was finally diagnosed as single infection with *M. avium*; antibiotic regimen was accordingly changed to RFP, EB, and clarithromycin. Four months after the initiation of treatment, her clinical condition remained stable.

## Discussion

Pleural effusion is a rare manifestation of NTM infection, whose frequency has been reported as 1.4% [1]. There has been only one case of NTM pleuritis in which diagnostic thoracoscopy was performed; macroscopic findings of the pleura were normal, and pathological diagnosis was just non‐specific lymphocytic pleuritis [4]. In the present case, epithelioid cell granulomas were observed in the biopsied nodule. As granulomatous nodules of the pleura can be identified in most patients with tuberculous pleurisy [5], we speculate that epithelioid cell granulomas were suggestive of NTM infections as well as tuberculosis. As far as we are aware, the present case is the first reported NTM pleurisy with *M. avium*‐positive nodular lesions of the pleura.

Culture of biopsy samples of the pleura was positive for *M. avium* both in the previous case and our own, while neither of them failed to demonstrate any pathogens from pleural fluid [4]. The proportion of culture‐positive pleural effusions among patients with NTM pleuritis is unknown; however, it is suspected to be not high enough, as the frequency of positive cultures from pleural fluid is only 15% among tuberculous pleuritis. By contrast, cultures of biopsy specimens are positive in 55–80% of such patients [6]. We postulate that pleural biopsy specimens should be cultured to establish a definitive diagnosis.

In conclusion, physicians should be aware of the possibility of NTM in differential diagnosis of pleural effusion. As culture of pleural fluid is not a strong tool for detecting NTM pathogens, demonstration of pleural nodules during thoracoscopy can contribute to prompt and accurate diagnosis and treatment of NTM pleuritis.

### Disclosure Statement

Appropriate written informed consent was obtained for publication of this case report and accompanying images.
